# Effect of thickened liquids in facilitating full oral nutrition for the preterm infant struggling to achieve full oral feeds at term postmenstrual age

**DOI:** 10.1038/s41372-025-02529-1

**Published:** 2025-12-22

**Authors:** Irena K. Wilson, Gregory P. Jansen, Alicia Hofelich Mohr, Michael Georgieff, Sara Ramel, Kelly Dietz, Holly Shifsky, Katlyn E. McGrattan

**Affiliations:** 1 Department of Speech-Language-Hearing Sciences, University of Minnesota, Minneapolis, MN USA; 2 Department of Neonatology, University of Minnesota, Minneapolis, MN USA; 3 Department of Radiology, University of Minnesota, Minneapolis, MN USA; 4 Department of Rehabilitation, Masonic Children’s Hospital, Minneapolis, MN USA

**Keywords:** Medical imaging, Paediatrics, Outcomes research

## Abstract

**Objective:**

Test the effect of thickened liquids on preterm infants (25–35 weeks of gestation) who are struggling to achieve full oral feeds.

**Study design:**

Retrospective case-control study of preterm infants struggling to achieve full oral feeds at term, prompting provision of thickened liquids without aspiration on instrumental assessment, and controls, matched for gestational age and comorbidities, who followed a typical thin liquid feeding progression. Paired t-tests were used to test differences in overall milk transfer (%, milk consumed/prescribed) before and after thickening, with mixed linear regression to test differences between the control and thickened group.

**Result:**

38 infants (19 thickened) were included. Prior to thickening, infants struggling with feeds had significantly slower rates of improvement in overall transfer than controls (struggling feeders, 1.3%/day; controls, 4.5%/day) (*p* < .001). Overall transfer increased 13% 48 h after thickening (*p* = 0.01).

**Conclusion:**

Thickened liquids may be an effective treatment for select preterm infants who are not progressing to full oral feeds at term postmenstrual age, even if aspiration is not observed on instrumental assessment.

## Introduction

Nearly all premature infants will suffer from feeding deficits at some point in their hospital stay [1]. The most common manifestations of these deficits include insufficient arousal to consume full oral nutrition [2, 3] and impaired coordination between sucking, swallowing, and respiration, resulting in apnea during oral feeding attempts [4–7]. For the majority of preterm infants, neuromuscular maturation will lead to the resolution of these impairments and obtainment of full oral nutrition by 36 weeks postmenstrual age (PMA) [8]. However, the persistence of feeding deficits among 20% of preterm infants will require continued hospitalization while they struggle to achieve this final developmental milestone [8].

With neonatal intensive care unit (NICU) stays costing approximately $3700/day in the United States[9] and families eager to be united at home, it is common practice to pivot from the “wait and see” approach, which relies on non-invasive assessments and treatments to manage preterm feeding deficits, to the use of instrumental assessments, such as the videofluoroscopic swallow study (VFSS), to explore if aspiration is causing these clinical symptoms at term PMA. Although the VFSS allows for visualization of physiology not otherwise observable to the naked eye, it does so at the cost of exposing infants to ionizing radiation, which carries long-term carcinogenic effects [10, 11]. In an effort to reduce radiation exposure, exams are limited in fluoroscopic visualization to just a sample of swallows, typically including less than 15% of an infant’s typical bottle feed, from which clinicians must make recommendations for the optimal feeding regimen [12].

Although observations of aspiration on the VFSS often lead clinicians to recommend more invasive treatment options, such as thickened liquids, in other cases, clinically symptomatic infants do not exhibit aspiration on the instrumental exam. Determination of whether these “normal” results are strictly a reflection of the sampling limitations inherent to the VFSS, in which case a treatment such as thickening may be beneficial, or truly reflect intact swallowing physiology, can be a daunting task. Thickened liquids have many negative side effects, including reduced nutrient absorption [13–15] and the need to switch the infant from breast milk to formula to facilitate effective thickening [16]. This makes weighing the risks and benefits critical in guiding care. Interestingly, previous retrospective investigations conducted on heterogeneous samples of dysphagic infants suggest thickened liquids may improve clinical symptoms regardless of whether aspiration is observed on VFSS [17, 18]. To our knowledge, there have not been any investigations examining the effectiveness of this practice on preterm infants struggling to achieving full oral feeds during their NICU stay. This investigation takes the first step in filling this void by examining the effect of thickened liquids on feeding performance among preterm infants who had clinical dysphagia symptoms but did not aspirate on VFSS. Our hypothesis is that select infants would improve with thickened liquids despite not aspirating on their VFSS exam.

## Materials and methods

### Participant identification

We conducted a retrospective case-control investigation examining feeding and systemic health parameters among two cohorts of preterm infants (<37 weeks gestation) during their NICU stay between 2019 and 2024 at Masonic Children’s Hospital. The first cohort included (1) preterm infants who struggled to achieve full oral feeds at term PMA and were not showing clinical indicators of an approaching resolution; (2) underwent a VFSS to evaluate for the source of impairments and did not exhibit aspiration of thin liquids on their exam, but (3) were provided thickened liquids in effort to remediate clinical symptoms (thickened group). As part of standard clinical care, all infants receiving care at the NICU where this investigation occurred received daily feeding assessments by a Feeding Therapist with specialized training in neonatal feeding. This included feeding readiness and quality scores, signs of physiologic stress, and overall transfer. Determination of when to conduct a VFSS followed an interdisciplinary discussion regarding the infant’s performance in the aforementioned metrics relative to comorbidities and the infant’s gestational and PMA. Infants were excluded if they were diagnosed with a syndrome or genetic anomaly associated with dysphagia, though those with common comorbidities of prematurity (e.g., bronchopulmonary dysplasia) were included. Swallow studies were conducted with Varibar^®^ barium contrast using the infant’s typically used bottle nipple, position, and compensatory strategies (e.g., pacing) by the infant’s feeding therapist and radiologist. All exams tested changes in swallowing over time by turning fluoroscopy on and off periodically as the infant continued to feed, though the timing of when this occurred was variable. The second “control” cohort included age and comorbidity-matched infants who followed the typical preterm feeding course, achieving full oral feeds without undergoing a VFSS or receiving thickened liquids.

### Data collection

Eligible infants underwent a medical record review to gather information, including sex, demographics, gestational age, birth weight, comorbidities, and characteristics of their feeding progression, such as formula type and caloric density, the date full oral feeds were achieved, and daily overall transfer. Overall transfer is the percent of the prescribed daily milk volume that the infant consumed by mouth. For example, if they were prescribed 520 mL/day, and consumed 229 mL orally with the rest gavage, that would equate to 44% overall transfer. Charts of infants in the thickened cohort were also reviewed for specifications of the characteristics of the feeding symptoms they presented with, whether penetration was observed on the VFSS, as well as characteristics of the thickened liquids provided, including the type (rice or oatmeal), brand (e.g., Gerber), and amount of thickener used. At the medical center where exams were completed, judgments of penetration were made by a pediatric radiologist based on the presence of barium entering the laryngeal vestibule.

### Statistical analysis

Characteristics of the samples were summarized using descriptive statistics, including mean ± standard deviation and proportions (*N*). Independent t-tests, chi-square, and Fisher’s exact tests were used for comparison of sample characteristics such as gender, gestational age, PMA at oral feeding initiation, and comorbidities between the thickened and control samples. Paired t-tests were used to test for differences in overall transfer 48 h before and after thickening. Evaluation of feeding trajectories from the start of oral feeds to full oral intake among the control and thickened group was calculated using mixed linear models, with slopes for trajectories before and after thickening estimated separately in a piecewise approach. Differences in the estimated coefficients for feeding progression between groups were evaluated with t-tests. This study was approved by the Institutional Review Board at the University of Minnesota, with informed consent waived due to its retrospective nature.

## Results

### Demographics

Thirty-eight infants (female 60%, *N* = 23) were included in the investigation. The thickened group was composed of 19 infants with an average gestational age of 31.1 ± 5 weeks, birthweight of 1580 ± 760 g, and PMA of 41.4 ± 3 weeks at the start of thickening. The primary comorbidity was bronchopulmonary dysplasia (47%, *N* = 9), with two infants (10%) also having an atrial septal defect. The control group, by nature of age and comorbidity matched selection, exhibited no significant differences in gestational age (30.9 ± 3.5 weeks) or comorbidities (bronchopulmonary dysplasia, 47%, atrial septal defect, 10%), nor sex, birth weight, or age at initiation of oral feeds (*p* ≥ 0.9). Table 1 provides full demographic and background feeding characteristics of the thickened and control groups.

**Table 1:** Demographics and clinical characteristics of thickened and control groups.

	Thickened (*N* = 19)	Control (*N* = 19)	Statistic	*p*
Gestational age	31.1 ± 5	30.9 ± 3.2	*t* (36) = –0.12	0.91
Birth weight (g)	1520 ± 660	1580 ± 760	*t* (36) = –0.25	0.80
Female	14 (74%)	9 (47%)	*χ*^2^(1) = 1.76	0.18
Race			*χ*^2^(4) = 3.32*	0.51
Asian	1 (5%)	3 (16%)		
American Indian/Alaska Native	0 (0%)	1 (5%)		
Black	7 (36%)	4 (21%)		
White	8 (42%)	6 (32%)		
Comorbidities				
Bronchopulmonary dysplasia	9 (47%)	9 (47%)	*χ*^2^(1) = 0	1
Atrial septal defect	2 (11%)	2 (11%)	OR = 1	1
Initiation of oral intake (PMA)	35.8 ± 2.8	35.7 ± 3.1	*t* (36) = –0.08	0.94

Data reflects *N* (%) or Mean ± STD. Numbers do not add up to 100% due to some not being reported.

*Chi-squared estimates from a small sample; no difference when collapsed white vs non-white, *χ*^2^(1) = 0.0006, *p* = 0.98.

### Pre-thickening feeding characteristics

Infants who received a VFSS and subsequent thickening initiated oral feeds at an average of 35.8 ± 2.8 weeks PMA; however, after not making what was clinically determined to be age-appropriate oral feeding progression by term PMA, they underwent the VFSS (42.4 ± 3.0 weeks PMA) to look for a source of the clinical impairment. Control group infants similarly initiated oral feeds at 35.7 ± 3.1 weeks. The common underlying feeding presentation in question was insufficient volume of oral intake to meet nutritional needs (100%, *N* = 19), as characterized by infants only consuming an average of 43 ± 19% of their prescribed nutrition by mouth in the 48 h preceding their VFSS exam. Other reported symptoms included apnea and bradycardia with feeds (26%), inability to wean oxygen supports (26%), pulling away and arching during feeds (21%), and coughing with feeds (10%). This was in direct contrast to the control group, for whom a VFSS or thickening was not clinically indicated. Despite the thickened and control groups having similar medical backgrounds, infants who subsequently received thickened feeds due to plateaued feeding progressions exhibited significantly smaller daily gains in overall transfer prior to thickening (1.3 ± 0.8% increase/day) than their control counterparts (4.5 ± 2.0% increase/day) (*p* < 0.001). As a result, infants in the control group obtained full oral feeds (100% overall transfer) by an average of 38.5 ± 2.0 weeks PMA, at which point infants who subsequently received thickened feeds were only consuming 35.7 ± 28.2% overall transfer.

### VFSS findings

Based on variability in provider practice at the time the exams were conducted, half of the VFSS exams were completed at 30 pulses per second (52%, *N* = 10), with all others conducted at 15. All exams were conducted using the infant’s typically utilized feeding strategies while being presented with thin Varibar^®^ barium. Although by the nature of the inclusion criteria none of the infants aspirated on their exam, the majority of infants exhibited thin liquid penetration (73%, *N*=14).

### Thickening effect

Thickened liquids were formulated using infant formula that was warmed in a Medela^TM^ warmer and pulverized Gerber oatmeal cereal in all but two infants, who were thickened with rice cereal prior to the change in practice pattern (89%, *N* = 17). Pulverization was achieved by grinding a large batch of cereal grains manually using a mortar and pestle approach, and then storing them in an airtight container for easy feeder utilization. The cereal-to-formula ratio varied across infants, with all recommended mixing ratios undergoing IDDSI testing to confirm the thickness level prior to implementation. The majority of infants were prescribed the unit’s standard “mildly thick” formulation of 1 tsp cereal/20 mL formula (79%, *N* = 15) using a Dr. Brown’s™ or MAM™ Level 2 or 3 nipple.

**Fig. 1:**
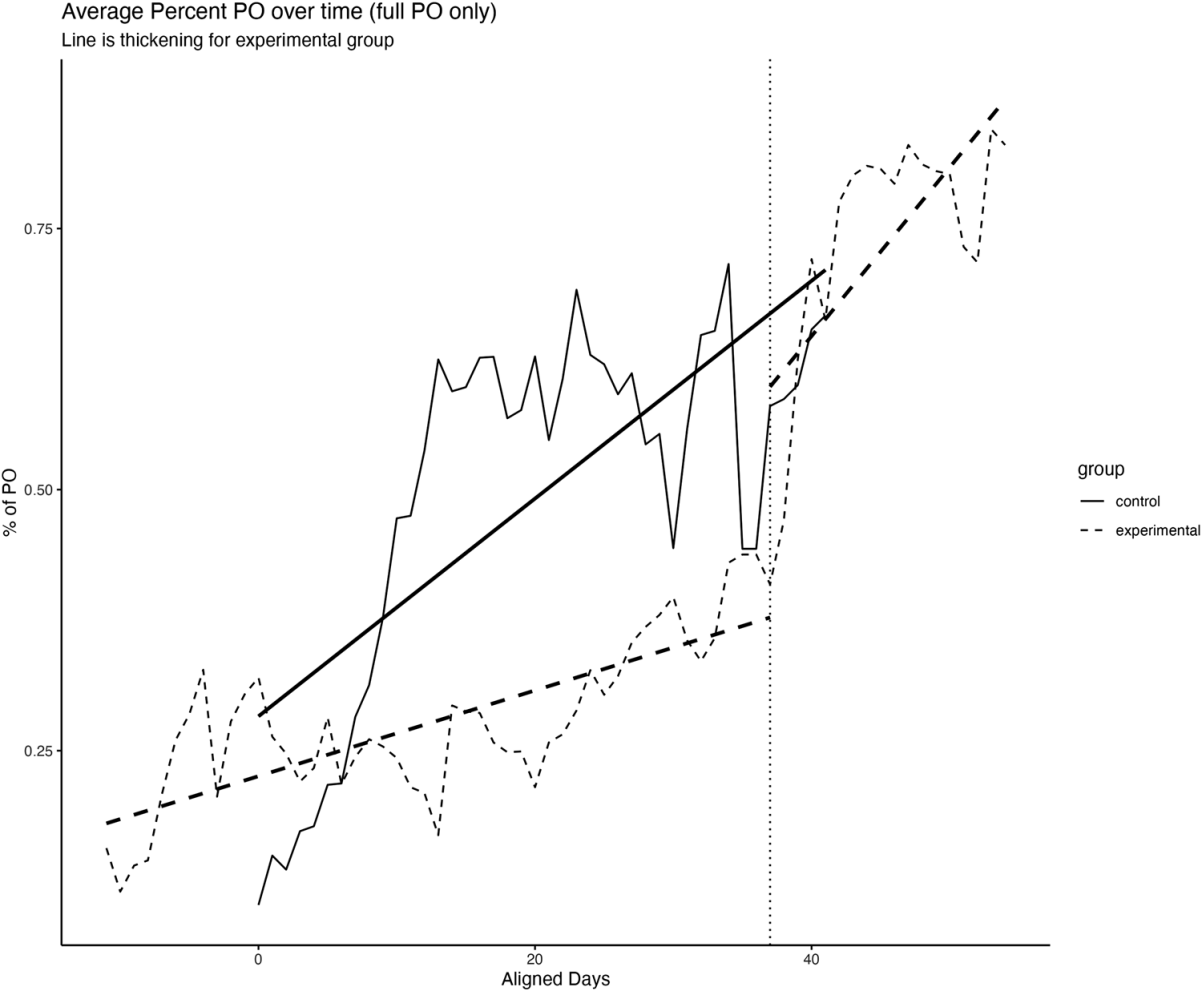
Trajectory of improvement in overall transfer from the start of full oral feeds to full oral feeds between the control infants (solid line) and those who struggled to achieve full oral feeds and later received thickened liquids (dashed line). Only those infants in the thickened group who achieved full oral feeds during their hospital stay are depicted. The vertical horizontal thickening line indicates the time at which thickening was initiated among the infants struggling to achieve full oral feeds.

Upon initiating thickened feeds, infants exhibited a 13.3% increase in overall transfer (*t* (18) = –2.84, *p* = 0.01). This was characterized by the consumption of 42.6 ± 19.1% of nutrition the 48 h preceding thickening, and the consumption of 55.9 ± 19.9% the 48 h following thickening. For 74% (*N* = 14) of infants, this post-thickening improvement in oral intake facilitated reaching full oral feeds at discharge an average of 8.2 ± 6.1 days later (42.4 ± 2.5 weeks PMA). The estimated daily gains in overall transfer after thickening (4.4%/day) were significantly faster than the gains that were achieved on thin liquids prior to thickening (1.3%/day)(comparison of slopes *t* (18) = 2.73, *p* = 0.006). Figure 1 depicts oral feeding trajectories of the control and treatment group pre and post-thickening.

Five (26%) of the infants in the thickening cohort did not achieve full oral feeds after the addition of thickened liquids and were discharged on alternative nutrition (e.g., NG tube or G-tube). All of these infants transitioned back to thin liquids at some point in their subsequent clinical course and progressed to achieve full oral feeds between 9 and 17 months old. There were no significant differences in pre-thickening oral feeding trajectories that differentiated those infants who discharged on full oral feeds of thickened liquids and those that did not (estimated difference of 0.5% a day, *t* = –0.99, *p* = 0.23). Table 2 provides a case-by-case listing of infant characteristics, VFSS results, and thickening outcomes within the sample.

**Table 2:** Characteristics of each individual included in the study.

Infant	GA	Birth weight *grams*	Comorbidities	Laryngeal Penetration	Thickening Cereal	Formula Type	Formula Caloric Density	Cereal Amount *per 20 mL*	Thickness	Bottle Nipple	Change in Overall Transfer^a^	Full Oral at Discharge
1	31w2d	1390	BPD	x	Rice	Similac Total Comfort	20 kcal/oz	1 tsp	Mildly	MAM 3	21.5%	
2	28w3d	1420	BPD	x	Oatmeal	Similac Special Care	20 kcal/oz	1 tsp	Mildly	MAM 3	43.2%	x
3	34w1d	2790		x	Oatmeal	Similac Advance	20 kcal/oz	1 tsp	Mildly	MAM 2	33.3%	x
4	31w1d	1860		x	Oatmeal	Nutramigen	22 kcal/oz	0.5 tsp	Slightly	MAM 2	-20.8%	
5	28w0d	1250	BPD	x	Oatmeal	Similac NeoSure	22 kcal/oz	1 tsp	Mildly	MAM 2	19.8%	x
6	32w0d	2120		x	Oatmeal	Breastmilk + Neosure	22 kcal/oz	1 tsp	Mildly	Dr. B 3	-10.6%	
7	32w0d	2010		x	Rice	Similac NeoSure	22 kcal/oz	1 tsp	Mildly	MAM 3	19.8%	
8	31w5d	1180		x	Oatmeal	Nutramigen	22 kcal/oz	1 tsp	Mildly	MAM 2	-15.5%	
9	34w3d	1560			Oatmeal	Similac NeoSure	22 kcal/oz	0.5 tsp	Slightly	MAM 2	-6.2%	
10	33w2d	2750		x	Oatmeal	Similac 360 Total Care	20 kcal/oz	1 tsp	Mildly	MAM 2	27.7%	x
11	34w0d	2010			Oatmeal	Breastmilk + NeoSure	26 kcal/oz	0.5 tsp	Slightly	MAM 1	-16.5%	x
12	33w4d	2970		x	Oatmeal	Similac 360 Total Care Sensitive	20 kcal/oz	1 tsp	Mildly	MAM 2	-5.0%	x
13	35w6d	2120		x	Oatmeal	Similac 360 Total Care	20 kcal/oz	1 tsp	Mildly	MAM 3	39.5%	x
14	32w3d	940	BPD		Oatmeal	Similac NeoSure	22 kcal/oz	1 tsp	Mildly	MAM 3	40.8%	x
15	27w1d	1050	BPD, ASD	x	Oatmeal	Similac NeoSure	20 kcal/oz	1 tsp	Mildly	MAM 2	26%	x
16	29w4d	870	BPD	x	Oatmeal	Similac Neosure	22 kcal/oz	0.5 tsp	Slightly	MAM 2	16%	x
17	25w3d	560	BPD, ASD		Oatmeal	Similac NeoSure	20 kcal/oz	1 tsp	Mildly	MAM 2	22.3%	x
18	25w3d	550	BPD	x	Oatmeal	Similac Special Care	20 kcal/oz	1 tsp	Mildly	Dr. B 3	9.5%	x
19	26w2d	630	BPD		Oatmeal	Similac NeoSure	20 kcal/oz	1 tsp	Mildly	MAM 2	8.7%	x

*GA* gestational age, *BPD* bronchopulmonary dysplasia, *ASD* atrial septal defect, *Dr. B* Dr. Brown’s™.

^a^Reflects change in overall transfer 48-h before and after thickening, with negative values indicating a reduction post thickening.

## Discussion

In this retrospective investigation, we examined the effect of thickened liquids on preterm infants who were not progressing to full oral feeds by term PMA, yet videofluoroscopic assessment did not indicate aspiration as an underlying source. Results suggest thickened liquids may be effective at facilitating full oral feeds for a subset of preterm infants exhibiting similar presentations, but not for all.

Although this is the first study to the author’s knowledge to examine the effect of thickened liquids without observed aspiration in preterm infants, previous investigators have reported similar beneficial results among other populations. In a retrospective investigation of a heterogeneous sample of infants who did not exhibit videofluoroscopic evidence of aspiration but did exhibit penetration, Duncan et al. found 91% of patients who were treated with thickened liquids had an improvement in symptoms [17]. Similarly, in a prospective investigation of term infants without comorbidities and documented penetration or aspiration on VFSS, Krummrich et al. found that both parents of infants with penetration and aspiration reported significant clinical benefits from thickened liquids, including taking larger volumes, reduced apnea episodes, congestion, coughing, feeding resistance, vomiting, and wheezing [18].

Though a future prospective investigation, where preterm infants without aspiration on VFSS are randomized to receive thickened liquids or remain on thin liquids, is necessary to draw larger conclusions about the true effects of this practice, the potential that thickened liquids could be beneficial even when VFSS results do not clearly indicate a deficit raises questions regarding the utility of the VFSS in preterm infants. It is well appreciated that the VFSS captures just a moment in time and does not reflect integrity throughout the entire bottle feed [12]. Further complicating this aspect of exam accuracy is the role of videofluoroscopic pulse rate. It has been postulated that pulse rates lower than 30 pulses per second may interfere with the ability to visualize events such as aspiration, even if fluoroscopy is being run when it occurs [19, 20]. Lastly, infants can be extremely variable in performance across feeds, demonstrating deficits on one feed, but not the next [21]. This leads to numerous variables that could potentially impede the ability to capture aspiration, even if it is the true underlying source for the infant’s clinical manifestation.

Although clinicians are well versed in these limitations and generally practice with appreciation that VFSS results must be extrapolated from beyond just the presence or absence of aspiration to guide clinical care, knowing precisely how to do this is another story. Visualizing aspiration lends to a much clearer-cut decision-making pathway directed towards treatment than observations of penetration, which often leave clinicians questioning whether treatment is indicated. This is largely because there have not been any systematic videofluoroscopic investigations elucidating “normal” swallowing characteristics in healthy, non-dysphagic infants. As such, clinicians are left to make decisions based on extrapolations from adult research elucidating normal swallowing physiology and pediatric research showing thickening treatment effects. Adult research suggests aspiration is rare, and likely indicative of an underlying impairment, while penetration is more common and may be a variant of normal [22]. If this is the case for infants, treatment in response to penetration on VFSS does not logically seem indicated. However, this is in direct conflict with the aforementioned pediatric research findings indicating beneficial thickening effects for infants who exhibit penetration [17, 18].

Results from our investigation further support the potential clinical relevance of penetration on VFSS, with 74% of the preterm infants in our investigation exhibiting penetration on VFSS. It is likely that the characteristics surrounding penetration, such as frequency and depth, may hold greater clinical precision in delineating normal variants from impairments. Miller et al. reported that the odds of thin liquid aspiration were four times higher among infants who exhibited frequent thin liquid penetration on the preceding swallows, and 46 times higher for those who exhibited thin liquid penetration to the level of the vocal folds [23]. Future investigations elucidating how this, and other clinical and instrumental parameters, can be used to better predict children who would benefit from interventions such as thickened liquids are necessary to better delineate treatment plans.

While limitations in our ability to fluoroscopically capture aspiration may be one source for the observed discrepancy between assessment findings and treatment effects, another source may be that the underlying etiology for the feeding impairment does not reside in bolus airway entry at all. Thickened liquids are frequently used as a compensatory intervention in the presence of aspiration as they move through the pharynx at a slower rate [24]. This enables the intricate series of muscular movements that facilitate swallowing to be executed with greater precision in isolation, and in coordination with each other [24]. In the case of aspiration, this is targeted at facilitating more time for hyolaryngeal elevation and anterior excursion to close off the laryngeal vestibule. However, these beneficial effects have also been reported in facilitating coordination between other physiologic processes of swallowing, such as the movements of the tongue base and soft palate [24]. Discoordination between oropharyngeal movement patterns critical to feeding is a well-established problem in the preterm infant [24, 25], though precisely how this discoordination influences the infant’s ability to consume full oral nutrition is not well understood. Expanding on this premise, another possible source for our treatment effects beyond the oropharynx may be in the treatment of gastroesophageal reflux, for which thickened liquids are a well-established treatment with demonstrated benefit for reducing regurgitation and vomiting episodes [12–14] as well as a reduction in parent-reported symptoms [15–17]. Overall, successful obtainment of oral nutrition requires integrity within a wide array of physiologic processes spanning the upper and lower aerodigestive tract. Future randomized controlled trials that test the thickened liquid effect and evaluate for the underlying etiology of functional feeding impairments are necessary to establish clinical care guidelines that balance the facilitation of oral feeding milestones and nutritional considerations.

Though there is building evidence that thickened liquids can provide beneficial treatment effects, there is an equally large, growing body of evidence demonstrating the many harmful side effects that come with them as well. At the forefront of these negative effects is the alteration to the nutritional profile of milk [16], with thickened liquids adding empty calories and trapping essential nutrients from being absorbed [13, 14]. Thickeners also alter milk’s osmolality, which can delay gastric emptying and intestinal transit [26]. Such concerns are of particular importance in consideration of their use in premature infants who are at increased risk for necrotizing enterocolitis, with thickeners attributed as a potential source [27]. A particularly troublesome result of the initiation of thickened liquids is that they often require infants to transition off breast milk to infant formula as enzymatic activity within breastmilk can break down the thickener and make them ineffective [28, 29]. This has tremendous implications given the known systematic benefits of breastmilk. Unfortunately, despite the many negative health effects, infants are frequently discharged on thickened liquids without a clear-cut plan or clinical support team to guide the family in the timing and methods of weaning their use. While investigation of these components of thickening was outside the scope of the current investigation, consideration of these critical side effects warrants future interdisciplinary investigation and the development of consensus guidelines for when and how thickening should be implemented in clinical care.

Results from this investigation should be interpreted with consideration of several limitations, starting with the retrospective study design. Although all efforts were made to control variables that may contribute to the observed findings, such as gestational age, comorbidities, and the natural course of preterm feeding progression, a true demonstration of treatment effect warrants a randomized control study design. As such, the observed associations should be interpreted cautiously. Another limitation pertains to the fluoroscopic sampling at 15 pulses per second for nearly half of the videofluoroscopic exams. It is possible that this lower pulse rate may have impeded the ability to identify clinically significant deficits, such as aspiration, that would more traditionally call for the treatment through thickened liquids [20]. Another variable that could have contributed to our findings is the change in milk flow rate resulting from the use of faster-flowing nipples. While it’s common practice to increase nipple flow rate when infants are placed on thickened liquids to enable milk extraction, there is no precise algorithm guiding which bottle nipple will allow the infant to extract the bolus at the same milk flow rate as they did on thin. It’s plausible that resulting changes in milk flow rate had a role in improving oral intake. Lastly, while the majority of infants were thickened to mildly thick liquids using a standard thickening recipe, a subset was thickened to slightly thick. Future prospective randomized controlled trials using standardized thickening amounts are necessary to further elucidate the effect of such practice.

## Conclusions

Thickened liquids may be an effective treatment for select preterm infants who are not progressing to full oral feeds at term PMA, even if aspiration is not observed on instrumental assessment. Future prospective, randomized controlled trials that also consider the negative nutritional implications of this practice are necessary to further evaluate treatment effect as well as specifications regarding its use.

## Data Availability

The datasets generated during and/or analysed during the current study are available from the corresponding author on reasonable request.

## References

[1] Jackson BN, Kelly BN, McCann CM, Purdy SC. Predictors of the time to attain full oral feeding in late preterm infants. Acta Paediatr. 2015;105:e1–e6. 26408819 10.1111/apa.13227

[2] white-Traut R, Berbaum M, Lessen B, McFarlin B, Cardenas L. Feeding readiness in preterm infants: the relationship between preterm behavioral state and feeding readiness behaviors and efficiency during transition from gavage to oral feeding. Am J Matern Child Nurs. 2005;30:52–9. 15622150

[3] Thoyre S, Shaker C, Pridham K. The early feeding skills assessment for preterm infants. Neonatal Netw. 2005;24:7–16. 15960007 10.1891/0730-0832.24.3.7 PMC2828611

[4] Vice FL, Gewolb IH. Respiratory patterns and strategies during feeding in preterm infants. Dev Med Child Neurol. 2008;50:467–72. 18422676 10.1111/j.1469-8749.2008.02065.x

[5] Lau C, Smith EO, Schanler RJ. Coordination of suck-swallow and swallow respiration in preterm infants. Acta Paediatr. 2003;92:721–7. 12856985

[6] Mizuno K, Ueda A. The maturation and coordination of sucking, swallowing, and respiration in preterm infants. J Pediatr. 2003;142:36–40. 12520252 10.1067/mpd.2003.mpd0312

[7] Gewolb IH, Vice FL. Maturational changes in the rhythms, patterning, and coordination of respiration and swallow during feeding in preterm infants. Dev Med Child Neurol. 2006;48:589–94. 16780629 10.1017/S001216220600123X

[8] Brumbaugh JE, Colaizy TT, Saha S, Van Meurs KP, Das A, Walsh MC, et al. Oral feeding practices and discharge timing for moderately preterm infants. Early Hum Dev. 2018;120:46–52. 29654994 10.1016/j.earlhumdev.2018.04.001 PMC5951763

[9] Valencia Z, Sen A, Martin K. NICU Admissions and Spending Increased Slightly from 2017–2021 https://healthcostinstitute.org/hcci-originals-dropdown/all-hcci-reports/nicu-use-and-spending-1#:~:text=As%20expected%2C%20average%20daily%20facility,for%20NICU%20Levels%20III%20admissions.2023.

[10] Weir K, McMahon S, Long G, Bunch J, Pandeya N, Coakley K, et al. Radiation doses to children during modified barium swallow studies. Pediatr Radiol. 2007;37:283–90. 17216172 10.1007/s00247-006-0397-6

[11] Weir K, McMahon S, Barry L, Masters IB, Chang AB. Clinical signs and symptoms of oropharyngeal alspiration and dysphagia in children. Eur Respir J. 2009;33:604–11. 19010985 10.1183/09031936.00090308

[12] McGrattan KE, McGhee HC, McKelvey KL, Clemmens CS, Hill EG, DeToma A, et al. Capturing infant swallow impairment on videofluoroscopy: timing matters. Pediatr Radiol. 2020;50:199–206. 31650190 10.1007/s00247-019-04527-w PMC7685400

[13] Bosscher D, Van Aillie-Bertrand M, Deelstra H. Effect of thickening agents, based on soluble dietary fiber, on the availability of calcium, iron, and zinc from infant formulas. Nutrition. 2001;17:614–8. 11448582 10.1016/s0899-9007(01)00541-x

[14] Bosscher D, Van Caillie-Bertrand M, Deelstra H. Do thickening properties of locust bean gum affect the amount of calcium, iron and zinc available for absorption from infant formula? In vitro studies. Int J Food Sci Nutr. 2003;54:261–8. 12850887 10.1080/09637480120092080

[15] Bosscher D, Van Caillie-Bertrand M, Van Dyck K, Robberecht H, Van Cauwenbergh R, Deelstra H. Thickening infant formula with digestible and indigestible carbohydrate: availability of calcium, iron, and zinc in vitro. J Pediatr Gastroenterol Nutr. 2000;30:373–8. 10776946 10.1097/00005176-200004000-00005

[16] Duncan DR, Larson K, Rosen RL. Clinical aspects of thickeners for pediatric gastroesophageal reflux and oropharyngeal dysphagia. Curr Gastroenterol Rep. 2019;21:30. 31098722 10.1007/s11894-019-0697-2 PMC9733977

[17] Duncan R, Larson K, Davidson K, May K, Rahbar R, Rosen RL. Feeding interventions are associated with improved outcomes in children with laryngeal penetration. J Pediatr Gastroenterol Nutr. 2019;68:218–24. 30320668 10.1097/MPG.0000000000002167 PMC6501833

[18] Krummrich P, Kline B, Krival K, Rubin M. Parent perception of the impact of using thickened fluids in children with dysphagia. Pediatr Pulmonol. 2017;51:1486–94. 10.1002/ppul.23700 28436603

[19] Bonilha HS, Wilmskoetter J, Tipnis SV, Martin-Harris B, Huda W. Estimating thyroid doses from modified barium swallow studies. Health Phys. 2018;115:360–68. 30045116 10.1097/HP.0000000000000890 PMC6634296

[20] Bonilha HS, Blair J, Carnes B, Huda W, Humphries K, McGrattan K, et al. Preliminary investigation of the effect of pulse rate on judgments of swallowing impairment and treatment recommendations. Dysphagia. 2013;28:528–38. 23559454 10.1007/s00455-013-9463-z PMC3762944

[21] McGrattan KE, Mohr AH, Weikle E, Hernandez K, Walsh K, Park J, et al. Establishing normative values for healthy term infant feeding performance: neonatal eating assessment tool-mixed, oral feeding scale, and early feeding skills assessment. Am J Speech Lang Pathol. 2023;32:2792–801. 37682537 10.1044/2023_AJSLP-22-00372

[22] Garand KLF, Hill EG, Amella E, Armeson K, Brown A, Martin-Harris B. Bolus airway invasion observed during videofluoroscopy in healthy, non-dysphagic community-dwelling adults. Ann Otol Rhinol Laryngol. 2019;128:426–32. 30700098 10.1177/0003489419826141 PMC7658791

[23] Miller AL, Miller CK, Fei L, Sun Q, Willging JP, de Alarcon A, et al. Predictive value of laryngeal penetration to aspiration in a cohort of pediatric patients. Dysphagia. 2024;39:33–42. 37243730 10.1007/s00455-023-10589-8

[24] Goldfield EG, Smith V, Buonomo C, Perez J, Larson K. Preterm infant swallowing of thin and nectar-thick liquids: changes in lingual-palatal coordination and relation to bolus transit. Dysphagia. 2013;28:234–44. 10.1007/s00455-012-9440-y PMC3638056 23274694

[25] Rommel N, van Wijk M, Boets B, Hebbard G, Haslam R, Davidson G, et al. Development of pharyngo-esophageal physiology during swallowing in the preterm infant. Neurogastroenterol Motil. 2011;23:e401–8. 21827583 10.1111/j.1365-2982.2011.01763.x

[26] Levy DS, O’sborn E, Hasenstab KA, Nawaz S, Jadcherla SR. The effect of additives for reflux or dysphagia management on osmolality in ready-to-feed preterm formula: practice implications. J Parenter Enter Nutr. 2018;43:290–7. 10.1002/jpen.1418 PMC6328349 29992586

[27] Woods CW, Robinson MJ. Development of necrotizing enterocolitis in premature infants receiving thickened feeds using SimplyThick. J Perinatol. 2012;32:150–2. 22289705 10.1038/jp.2011.105

[28] Almeida MBDM, Gomes Júnior SC, Silva JBD, Silva DAD, Moreira MEL. Study on viscosity modification of human and formula milk for infants with dysphagia. Rev CEFAC. 2017;19:683–9.

[29] Brooks L, DiStefano CC, Clayton H, Gethers CT. Thickening human milk: the effect of time, temperature, and thickener for infants with dysphagia. Eur J Pediatr. 2024;183:1839–48. 38277000 10.1007/s00431-024-05434-5

